# A Scabies Mite Serpin Interferes with Complement-Mediated Neutrophil Functions and Promotes Staphylococcal Growth

**DOI:** 10.1371/journal.pntd.0002928

**Published:** 2014-06-19

**Authors:** Pearl M. Swe, Katja Fischer

**Affiliations:** QIMR Berghofer Medical Research Institute, Infectious Diseases Program, Biology Department, Herston, Brisbane, Australia; National Institute of Allergy and Infectious Diseases, National Institutes of Health, United States of America

## Abstract

**Background:**

Scabies is a contagious skin disease caused by the parasitic mite *Sarcoptes scabiei*. The disease is highly prevalent worldwide and known to predispose to secondary bacterial infections, in particular by *Streptococcus pyogenes* and *Staphylococcus aureus*. Reports of scabies patients co-infected with methicillin resistant *S. aureus* (MRSA) pose a major concern for serious down-stream complications. We previously reported that a range of complement inhibitors secreted by the mites promoted the growth of *S. pyogenes*. Here, we show that a recently characterized mite serine protease inhibitor (SMSB4) inhibits the complement-mediated blood killing of *S. aureus*.

**Methodology/Principal Findings:**

Blood killing of *S. aureus* was measured in whole blood bactericidal assays, counting viable bacteria recovered after treatment in fresh blood containing active complement and phagocytes, treated with recombinant SMSB4. SMSB4 inhibited the blood killing of various strains of *S. aureus* including methicillin-resistant and methicillin-sensitive isolates. Staphylococcal growth was promoted in a dose-dependent manner. We investigated the effect of SMSB4 on the complement-mediated neutrophil functions, namely phagocytosis, opsonization and anaphylatoxin release, by flow cytometry and in enzyme linked immuno sorbent assays (ELISA). SMSB4 reduced phagocytosis of *S. aureus* by neutrophils. It inhibited the deposition of C3b, C4b and properdin on the bacteria surface, but did not affect the depositions of C1q and MBL. SMSB4 also inhibited C5 cleavage as indicated by a reduced C5b-9 deposition.

**Conclusions/Significance:**

We postulate that SMSB4 interferes with the activation of all three complement pathways by reducing the amount of C3 convertase formed. We conclude that SMSB4 interferes with the complement-dependent killing function of neutrophils, thereby reducing opsonization, phagocytosis and further recruitment of neutrophils to the site of infection. As a consequence secreted scabies mites complement inhibitors, such as SMSB4, provide favorable conditions for the onset of *S. aureus* co-infection in the scabies-infected microenvironment by suppressing the immediate host immune response.

## Author Summary

There is increasing evidence that in the tropics bacterial pyoderma and life-threatening downstream complications caused by *Staphylococcus aureus* and *Streptococcus pyogenes* are often linked with scabies infestation. The bacteria were commonly thought to find easy entry into the skin, damaged by scabies mites. However, there may be more to it. Our study aimed to identify the molecular mechanisms underlying the link between mites and bacteria. We recently characterized a molecule (SMSB4) that the mite produces for self-protection from the immediate host defense system in the skin. We show here that SMSB4 reduces the uptake of *S. aureus* by neutrophils, which are the killer immune cells, first to arrive at the infection site. These cells are guided by a number of complement factors. SMSB4 reduces the deposition of several complement components on the bacteria surface, thereby interrupting the essential defense cascades and inhibiting further neutrophil recruitment to the site. In summary, a mite molecule that likely originally evolved to protect the mite against the host defense plays a further role in the pathogenesis of secondary infections: when secreted into the damaged skin it promotes the onset of *S. aureus* infection thereby increasing the prevalence of debilitating disease associated with scabies.

## Introduction

Scabies is a ubiquitous and contagious skin infection caused by a burrowing parasitic mite *Sarcoptes scabiei*. In addition to the morbidity associated with the typical intense itching; a rather more important significance of scabies are the associated secondary bacterial infections, which predispose the host to serious downstream systemic and life-threatening diseases [1]. Scabies is one of the most common dermatological conditions on a global scale with disease burden of up to 70% in rural India, between 18 and 42% in the South Pacific, and erratic reports from Africa and South America [2], [3]. In Australia, prevalence of scabies is particularly high in the remote Aboriginal communities in the tropical north, with up to 70% of children present with scabies and pyoderma before reaching two years of age [4]. Community-wide treatment of scabies decreases pyoderma which suggests a key role of the burrowing mite [5], [6]. *Staphylococcus aureus* and *Streptococcus pyogenes* are the common causative agents of scabies associated pyoderma [7], [8]. The annual incidence of bacteremia due to *S. aureus* is six times higher in the Australian Aboriginal population than the rest of the Australian population [9]. More recently, methicillin resistant *S. aureus* (MRSA) has been reported in 64% of children presented with scabies in a regional hospital of North West Queensland [10]. While epidemiological evidence has been well-established that bacterial infections are closely linked with scabies, and mechanical damage of the upper epidermal layers by the mites is one obvious prerequisite, the relationship itself, between host, mites and bacteria, is poorly understood. Given that scabies is a primary risk factor for bacterial skin infections and increased incidence of MRSA infections, it is of increasing relevance to understand the molecular links in the trilateral relationship of host, mites and bacteria.

Upon entry, the pathogen is confronted by the complement system, which is part of the innate defense mechanism of the human host. Three different pathways initiate complement cascade, the classical (CP), lectin (LP) and alternative pathways (AP). The CP is initiated by binding of C1q to bacterial bound antibody or surface microbial sugar. This activates the protease C1r and then C1s, which cleaves C4 into C4a and C4b. C4b covalently binds to the microbial surface and is joined by C2, which is then cleaved by C1s to form an enzyme complex C3 convertase (C4b2a) [11]. The LP is initiated when microbial surface sugars are recognized by mannose binding lectin (MBL) or ficolins, and complexed with MBL-associated proteases (MAPSs-1,-2,-3), which are functional homologues of C1r and C1s [12]–[14]. In the AP, properdin recognizes the microbial sugars and initiates the *de novo* assembly of the AP C3 convertase (C3bBb) on the pathogen surface [15]–[18]. All three pathways converge in the formation of a key enzyme, the C3 convertase. This cleaves C3, releasing a small chemo attractant C3a, and C3b, which is deposited on the microbial surface [19]. C3b deposition is crucial for eradication of microbes as it marks the microbes for efficient uptake and subsequent killing by phagocytes. Moreover, at a high local concentration C3b binds to C3 convertase thereby turning into C5 convertase (C4b2a3b/C3bBb3b), which cleaves the complement protein C5 into C5a and C5b. C5a is a potent chemoattractant, which attracts neutrophils, monocytes and macrophages to the site of infection. C5b and further complement components C6, C7, C8 and C9 form the membrane attack complex (MAC/C5b-9) on the cell surface, causing direct cell lysis in sensitive cells [11].

*S. aureus* is a versatile pathogen, causing serious disease in humans. It has evolved various mechanisms to impair and evade the host immune system. It has been reported to secret a variety of host complement inhibitors that decrease C3b deposition on the microbial surface, e.g. staphylococcal complement inhibitor [20], extracellular fibrinogen-binding protein [21], staphylokinase [22] and aureolysin [23]. Interestingly, scabies mites are also known to excrete multiple families of complement inhibitors, which are secreted into the mites intestinal system and are thus believed to prevent damage of the mite gut [24]–[26]. They are excreted with the mite feces and have been localized in the mite burrows in the upper epidermis [24], [25]. We reported that scabies mite complement inhibitors promote the survival and growth of *S. pyogenes* in whole blood *in vitro* assays [26]. We proposed that phagocytosis by neutrophils was impeded through complement inhibition. One of the mite complement inhibitors, a 54 kDa serpin named SMSB4, displayed direct interactions with several complement proteins and interfered with the initial and progressing steps of the complement cascade [25]. However, the process had previously not been analyzed in the context of a bacterial surface and the involvement of neutrophils had not been demonstrated. In addition it was unknown whether the SMSB4-mediated inhibition of complement activation offers congenial conditions for other major skin bacterial pathogens, such as *S. aureus*. Here we report that SMSB4 also promotes the survival and growth of *S. aureus* by interfering with the initiation of complement activation, leading to a reduced opsonization and to a reduced phagocytosis and killing of bacteria by neutrophils. Thus we postulate that SMSB4 is a major player in the tripartite interactions between host, parasite and any opportunistic bacterial pathogen.

## Methods

### Ethics statement

Normal human serum (NHS) for complement activation assays and whole blood samples for bactericidal assays was prepared from blood donated by healthy volunteers. Informed written consent was obtained from all blood donors. The protocol was approved by the medical ethics committee of the QIMR Berghofer Medical Research Institute.

### Blood donation and screening

Informed consent was obtained from all blood donors. The protocol was approved by the medical ethics committee of the QIMR Berghofer Medical Research Institute. Among several donors tested in preliminary experiments, blood from all donors showed bactericidal effect against *S. aureus*. Blood from two donors was used in all further assays requiring fresh whole blood.

### Bacterial strains and growth conditions

*S. aureus* commercial strain Xen29 (Kan^R^) was purchased from Caliper Life Sciences Inc. (Massachusetts, USA) and this strain was used for all assays. *S. aureus* strains associated with pyoderma were provided by Dr. Deborah Holt at the Menzies School of Health Research, Charles Darwin University, Darwin. Isolates HS56 (MSSA), HS16 (MRSA) [27] are Panton-Valentine leukocidin positive, multi-locus sequence type 93 (commonly known as the Queensland clone) [9] while M5 (MSSA), M34 (MRSA) [28] belong to CC75, a genetically distinct type of *S. aureus* lacking staphyloxanthin [29]. All strains were routinely cultured at 37°C aerobically in either Tryptic soy broth (TSB; Thermo Fisher Scientific Pty. Ltd., Australia), shaking at 200 rpm or on Tryptic soy agar (TSA) plates.

### Preparation of cell suspensions

*S. aureus* cell suspensions were prepared from mid-log growth phase cultures (OD_600_ = 1.0). Cells were harvested by centrifugation (4000 ×*g*, 10 min, 4°C), washed twice in phosphate buffered saline (PBS) and resuspended to a final OD_600_ = 0.03 (∼5× 10^6^ cfu/ml) in the same buffer. The cell number of this suspension was enumerated by plate count of colony forming units (cfu/ml) on TSA at 37°C overnight.

### Production and purification of recombinant SMSB4

SMSB4 was cloned and expressed in *Escherichia coli* BL21 (Qiagen) and purified under denaturing condition as described previously by Mika *et. al.* [25], with minor modifications. Briefly, SMSB4 cDNA (Yv5004A04, GenBank accession no. JF317222) of the human scabies mites *S. scabiei* cloned into the pQE9 expression vector (Qiagen) was transformed into *E. coli* BL21. *E. coli* cells were cultivated in Luria broth (Becton Dickinson) containing 100 µg/mL ampicillin at 37°C over night. After inoculation in 2YT medium (Becton Dickinson) containing the same concentration of ampicillin, the cells were grown at 37°C, shaking at 200 rpm until an OD_600_ of 0.6–0.7 was reached. Expression of recombinant SMSB4 was induced by addition of 0.5 mM IPTG, and continued shaking at 200 rpm for a further 4 h. Cells were collected by centrifugation at 6000 ×*g* at 4°C for 20 min, resuspended in serpin buffer (50 mM Tris, pH 8.0, 100 mM NaCl, 10 mM EDTA, 1 mM PMSF) and lysed in 250 µg/ml lysozyme and 10 µg/ml DNase at room temperature (RT) under continuous rotation for 1 h. All of the following purification steps were performed at 4°C. After sonication of the spheroplasts by a Sonifier 250 (Branson), inclusion bodies were washed five times using serpinX buffer (50 mM Tris, pH 8.0, 100 mM NaCl, 10 mM EDTA, 0.5% (v/v) Triton X-100) and retrieved by centrifugation (16,000 ×*g* for 20 min at 4°C). The resulting pellet was dissolved in solubilization buffer (6 M guanidine hydrochloride, 50 mM Tris, pH 7.8, 1 mM DTT) for 1 h. Proteins were further purified by nickel affinity chromatography. Solubilized protein was diluted 1∶1 with bind buffer (6 M urea, 100 mM NaH_2_PO_4_, 10 mM Tris, pH 8.0, 5 mM imidazole, 150 mM NaCl, 1% (v/v) glycerol, 1 mM DTT) and bound overnight to a pre-equilibrated 1ml Ni-NTA matrix (Qiagen) in a PolyPrep column (BioRad) on a rotating shaker. The column was washed twice with 5 ml of wash buffer (6 M urea, 100 mM NaH_2_PO_4_, 10 mM Tris, pH 6.3, 5 mM imidazole, 150 mM NaCl, 1% (v/v) glycerol, 1 mM DTT). Bound proteins were eluted twice using 3 ml of elution buffer (6 M urea, 100 mM NaH_2_PO_4_, 10 mM Tris, pH 8.0, 250 mM imidazole, 150 mM NaCl, 1% (v/v) glycerol and 1 mM DTT). Purified recombinant proteins were refolded overnight by drop wise addition of the protein elution into refolding buffer (300 mM L-arginine, 50 mM Tris, 50 mM NaCl and 5 mM DTT, pH 10.5) using a Minipuls 3 pump (Gilson) at a flow rate of 20 µl/min under gentle stirring. Refolded proteins were concentrated using an Ultrasette Lab Tangential Flow Device (10 kDa MWCO, PALL Life Sciences), followed by further concentration in centrifugal filters (10 kDa MWCO, Amicon Ultra, Millipore). Protein concentrations were determined by Bradford protein assay (Bio-Rad) with bovine serum albumin (BSA) (Invitrogen) as a standard according to the manufacturer’s instructions. Molecular mass and purity were confirmed using SDS-PAGE analysis with Coomassie blue R-250 staining. For all assays, SMSB4 was buffer exchanged into the corresponding assay buffers using 0.5 ml centrifugal filters (10 kDa MWCO, Amicon Ultra, Millipore).

### Whole blood bactericidal assays

Bactericidal assays were performed with human whole blood collected in standard vacutainers containing hirudin at a concentration >15 µg/ml as anticoagulant (Dynabyte Informationssysteme GmbH, Munich, Germany). The assays were performed as described previously [26],_ENREF_6_ENREF_6_ENREF_6 with some modifications. Bacteria were grown overnight at 37°C with shaking at 200 rpm in 5 ml TSB. The overnight culture was diluted to an initial OD_600_ of 0.05 in a fresh aliquot of 5 ml THB and the *S. aureus* culture was grown to mid-log growth phase (OD_600_ 1.0) at 37°C with shaking at 200 rpm. This culture was diluted 2×10^3^ fold in PBS to obtain an approximately 1×10^5^ cfu/ml of *S. aureus* challenge dose. To 100 µl of human venous blood, either of the following compounds were added in a volume of 27.5 µl: purified recombinant SMSB4 in the experimental samples, cobra venom factor (CVF) in the positive controls, and BSA or GVB^2+^ buffer (5 mM veronal buffer, 140 mM NaCl, 0.1% (w/v) gelatin, 1 mM MgCl_2_, 0.15 mM CaCl_2_, pH 7.35) in the negative controls. Finally 12.5 µl of the simultaneously prepared *S. aureus* challenge suspension were mixed to a total volume of 140 µl. Samples were placed on a rotisserie and incubated with end over end mixing for 1–3 h at 37°C. Subsequently 50 µl aliquots from each appropriately diluted tube were plated in duplicate on TSA. The plates were incubated overnight at 37°C under aerobic conditions and bacterial numbers were enumerated as cfu/ml. Bacterial recovery was calculated as a percentage of the number of bacteria recovered from samples treated with various test compounds in reference to the *S. aureus* challenge dose.

### Preparation of Fluorescein Isothiocynate (FITC) labeled *S. aureus*

A *S. aureus* cell suspension was prepared from a mid-log growth phase culture resuspended in PBS to OD_600_ = 1.0 as described above. This suspension was incubated with 0.01% (v/v) final concentration of FITC solution (Sigma) at 37°C for 30 min with shaking at 200 rpm in a light protected environment. Cells were pelleted by centrifugation at 4000 ×*g*, 10 min, 4°C. Excess FITC was removed by washing twice with 0.1 M carbonate buffer (pH 9.6) and resuspended to OD_600_ = 0.1 in the same buffer. Fluorescence of *S. aureus* cells was examined under fluorescence microscope (Leica Inverted Fluorescence Microscope, Lecia Microsystems). Cells were aliquoted into 300 µl volume and stored at −80°C in the dark until use.

### Phagocytosis of *S. aureus*

Human neutrophils were purified by Histopaque -1077 (density 1.077, Sigma)/Histopaque -1119 (density 1.119, Sigma) gradient according to the manufacturer’s instructions using heparinized whole blood from a single donor. Blood was collected in standard BD vacutainers containing 150 USP units of sodium heparin (Becton Dickinson). The numbers of purified neutrophils were counted in a hemocytometer and purity was checked by flow cytometry (Becton Dickinson FACSCanto II). Fifty μl of 20% serum freshly prepared from the same blood donor was incubated with 50 µl of various concentrations of purified recombinant SMSB4 in PBS and 5 µl of FITC-*S. aureus*, at 37°C for 30 min in the wells of a round-bottom 96 well plate (Nunc). Samples with serum heated at 56°C for 30 min prior to the experiment were included as a negative control. Plates were centrifuged for 3 min at 500 ×*g*. Phagocytosis was initiated by the addition of 50 µl of purified neutrophils into the wells containing FITC-*S. aureus* to achieve a neutrophil to bacteria ratio of 10∶1 (e.g. 10^6^ neutrophils to 10^5^ bacteria). The plate was shaken on a horizontal shaker at 200 rpm at 37°C for either 40 min or various time points. Reactions were stopped by the addition of 50 µl of 4% ice cold paraformaldehyde (PFD) solution into the wells and the plate was incubated on ice for a further 30 min. Exogenous, non-phagocytosed *S. aureus* cells were removed by gently washing and aspirating the supernatant twice with 200 µl ice cold PBS (900 ×*g*, 3 min, 4°C). Adherent neutrophils that remained on the bottom of the wells were resuspended in 200 µl PBS and uptake of the FITC-*S. aureus* was evaluated by flow cytometry (FACSCanto II, Becton Dickinson) measuring the fluorescence of 10^6^ gated neutrophils.

### Complement depositions on *S. aureus* by ELISA

To each well of a 96-well assay plate (Maxisorp, Nunc), 100 µl of approximately 5.0×10^6^ cfu/ml of *S. aureus* cell suspension was added, incubated first at 37°C for 1 h and subsequently at 4°C overnight. Wells were washed 4 times with 250 µl PBS containing 0.05% Tween-20 in between each step of the assay. The wells were blocked with block buffer (4% BSA in PBS containing 0.05% Tween-20) for 4 h at RT. Meanwhile, 30 µl of 10% pooled human serum diluted in GVB^2+^ buffer (5 mM veronal buffer, 10 mM NaCl, 0.1% (w/v) gelatin, 1 mM MgCl_2_, 0.15 M CaCl_2_, pH 7.35) was incubated with 30 µl of SMSB4 at 37°C for 30 min in a V-shaped bottom 96-well plate (Nunc). Fifty μl of these mixtures were transferred to the 96-well plate coated with *S. aureus* that was before blocked with the blocking buffer. The plate was incubated at 37°C for further 30 min. Bound complement proteins were detected by incubation with 60 µl of primary antibodies against human complement factors for 1 h at RT. For immunodetection antibodies against C1q, C3d, C4c (Dako, Denmark), properdin (R&D systems) and SC5b-9 neoantigen-specific antibody recognizing the MAC complex (Complement Technology Inc., USA) were used at a dilution of 1∶4000, and against MBL (manufacturer) at dilution of 1∶1000. The wells were subsequently incubated with 60 µl of horseradish peroxidase (HRP)-conjugated goat anti-rabbit or HRP-conjugated rabbit anti-goat secondary antibodies (Dako, Denmark) at dilutions of (1∶1000-1∶4000 in block buffer) at RT for 30 min-1 h, depending on the primary antibody specificity and signal intensity. The assay was developed with 60 µl of OPD reagent (Dako, Denmark) containing 0.01% hydrogen peroxide by incubation at RT until serum only positive control turned yellow. Reactions were stopped by addition of 50 µl of 0.5 N H_2_SO_4_ and absorbance was measured at OD_490_ on a POLARstar Optima fluorescent microtiter plate reader (BMG Labtech, Melbourne, Australia). The absorbance obtained in the serum only sample was defined as 100%.

### Statistical analysis

Statistical significance was determined using 2way ANOVA, either Tukey’s or Sidak’s multiple comparisons test (GraphPad Prism software, version 6.0; GraphPad Software Inc. USA). Values of p<0.05 were considered significant.

## Results

### SMSB4 reduces blood killing of *S. aureus*

**Figure 1:**
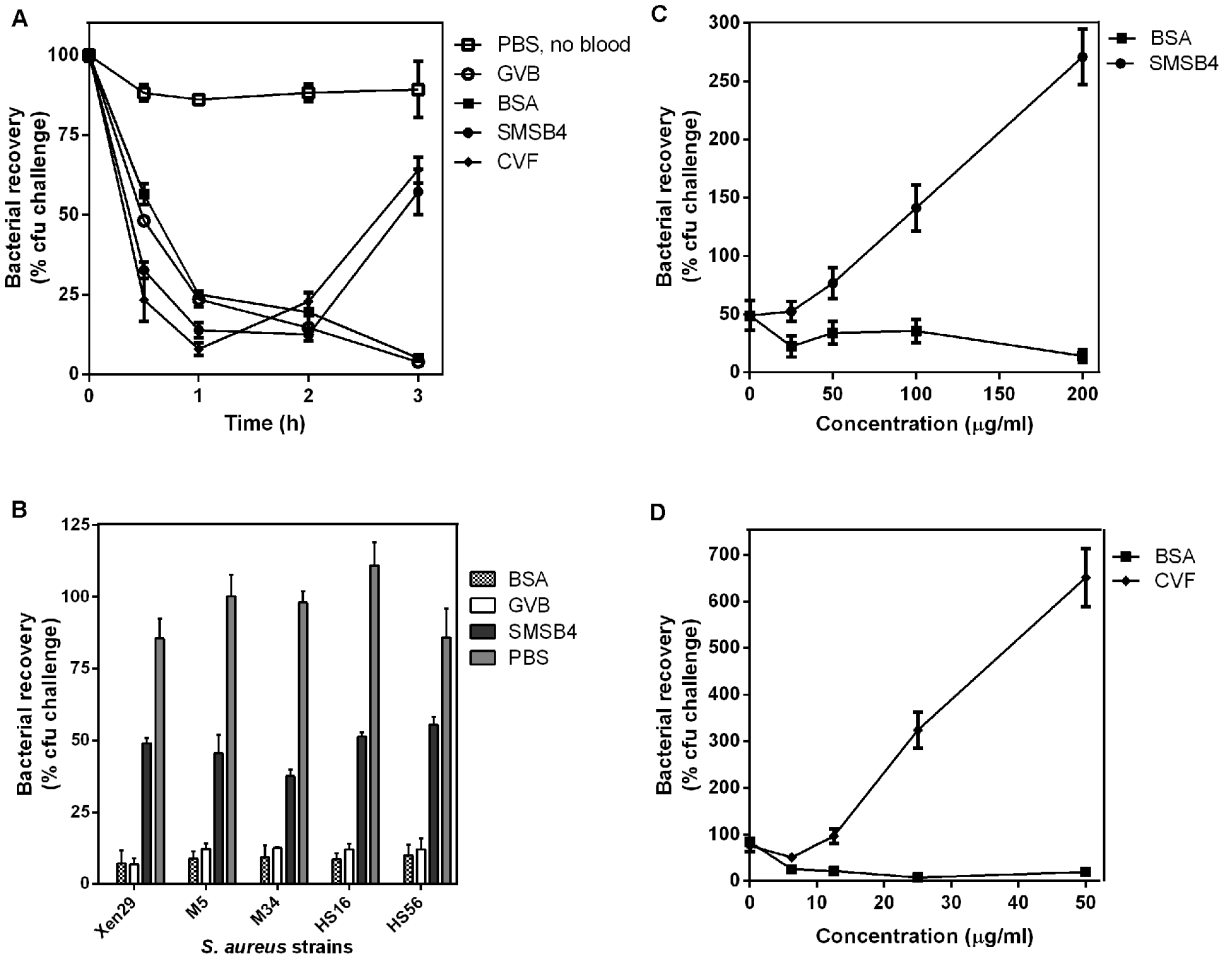
SMSB4 reduces the blood killing of *S. aureus* strain Xen29 in whole blood (A) and pyoderma isolates of *S. aureus* (B). SMSB4 promotes bacteria growth in a concentration dependent manner (C) similarly to CVF (D). *S. aureus* Xen29 or pyoderma isolates MRSA strains (HS16, M34), MSSA strains (HS56, M5) were harvested from mid-log growth phase culture. Bacteria (1×10^5^ cfu/ml) were challenged with whole blood pre-treated with either 100 µg/ml SMSB4, positive control 10 µg/ml CVF, negative controls 100 µg/ml BSA or GVB^2+^ buffer only. *S. aureus* cells in PBS only without blood was also included to illustrate that the reduction in bacteria number was due to blood killing (**A**). Numbers of bacteria were counted as cfu/ml at various time points (**A**) or at 3 h (**B**, **C**, **D**). Bacterial recovery was calculated as a percentage of the challenge dose. Results are shown as means ± SEM from three independent experiments. The statistical significance of differences between samples was estimated using two way ANOVA with Tukey’s multiple comparison test. **, *p*<0.01; ***, *p*<0.001; ****, *p*<0.0001, ns, not significant (B).

To investigate the effect of SMSB4 on the onset of *S. aureus* infection under physiological conditions, we performed bactericidal assay in SMSB4 treated blood when challenged with a known number of *S. aureus* Xen29. Samples treated with GVB^2+^ buffer or BSA were included as negative controls and samples treated with a known complement inhibitor, CVF as a positive control. We demonstrated that *S. aureus* was killed in blood when treated with GVB^2+^ buffer or 100 µg/ml of BSA, as up to 90% reduction in cfu/ml was seen after 3 h (Figure 1a). In contrast, bacteria numbers began to recover after 2 h in the samples treated with 100 µg/ml of SMSB4 and 10 µg/ml of CVF, approximately 50% increase in recovery at 3 h compared with the initial time points (Figure 1a). Bacterial recovery remained unchanged over time in the samples incubated with PBS only instead of blood, indicating that reduction in *S. aureus* number was due to blood killing.

Since complement-mediated blood killing is a generalized protective host response to an infection, we tested this bactericidal effect on various skin isolates of *S. aureus* including MRSA and MSSA phenotypes. The level of blood killing observed when MRSA strains (HS16, M34) and MSSA strains (HS56, M5) tested was very similar to what was seen for the type strain Xen29 (Figure 1b). In samples treated with either GVB^2+^ buffer or 100 µg/ml of BSA, the bacterial recovery ranged from 7–10% across all *S. aureus* strains tested (Figure 1b). A comparable degree of bacterial recovery (approximately 50%) was observed across all strains when treated with 100 µg/ml of SMSB4 (Figure 1b). We further established that SMSB4 reduced blood killing and promoted *S. aureus* growth in a dose dependant manner. A five-fold increase in bacterial recovery was observed in the presence of SMSB4 concentrations rising from 50–200 µg/ml (Figure 1c). A similar effect was observed when raising concentrations of the commercial complement inhibitor CVF were tested as a positive control (Figure 1d).

### SMSB4 reduces complement-driven neutrophil functions required for *S. aureus* killing

#### (i) SMSB4 reduces phagocytosis

**Figure 2:**
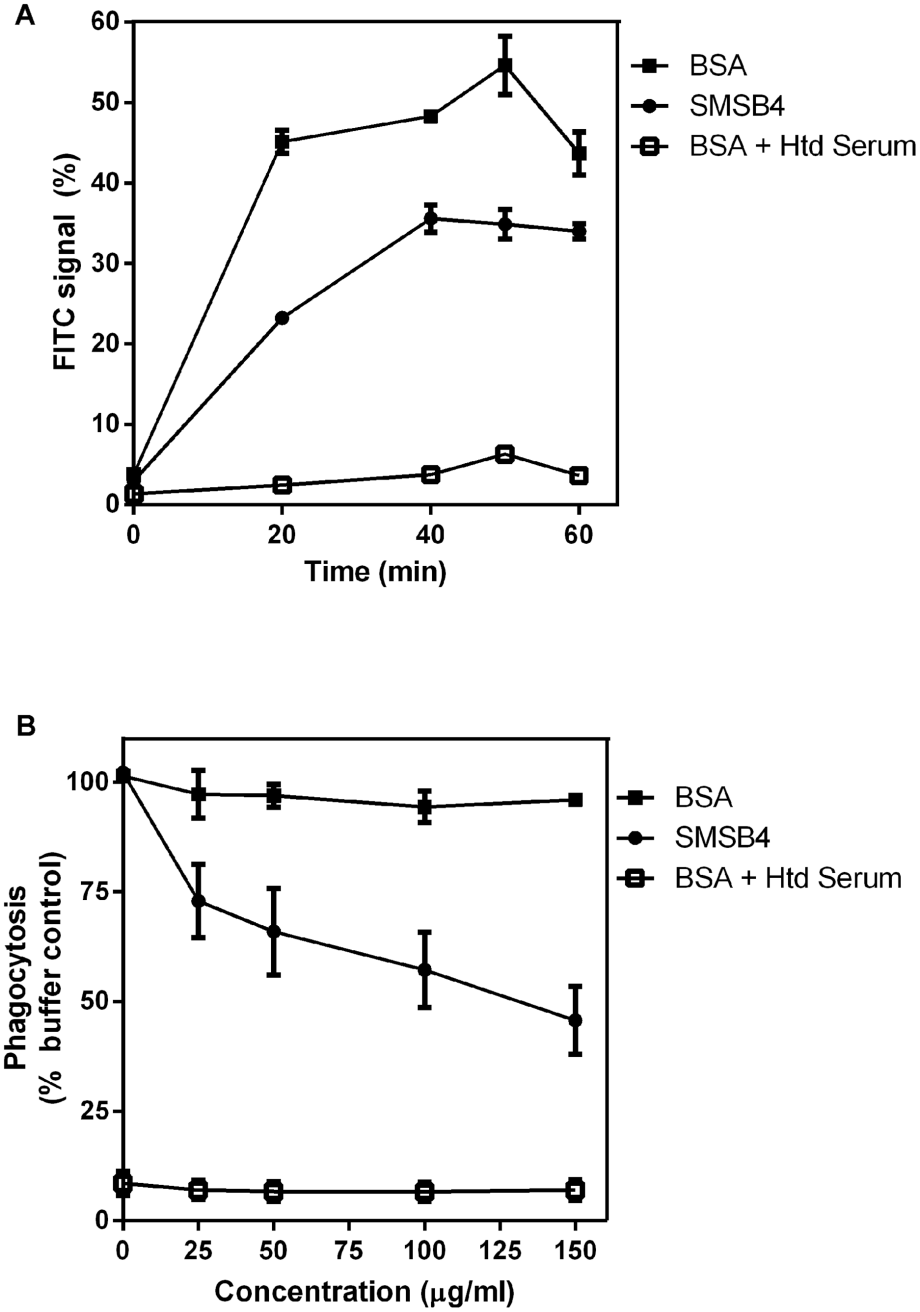
SMSB4 reduces phagocytosis of *S. aureus* by neutrophils. FITC-labeled bacteria (10^5^ cfu) was opsonized either with 20% NHS or heated serum and challenged with neutrophils (10^6^ cells). Serum samples were pre-treated for 30 min at 37°C with 50 µg/ml SMSB4, 50 µg/ml BSA (**A**), or various concentrations of SMSB4 or BSA (**B**). Uptake of FITC by neutrophils was measured at various time points over 1 h (**A**) or at 40 min (**B**). Results are shown as means ± SEM from three independent experiments.

While MAC complex is deposited on the surface of *S. aureus*, gram-positive bacteria such as *S. aureus* are insensitive to complement-mediated lysis by MAC formation [30]. Consequently the killing effect of blood in the bactericidal assays reported here is most likely due to neutrophil killing. We therefore investigated the processes essential for the complement-mediated killing function of neutrophils. Firstly, we tested the effect of SMSB4 on phagocytosis of *S. aureus* by purified human neutrophils. FITC-*S. aureus* were opsonized with serum pre-treated with SMSB4 or BSA, and human neutrophils were challenged with bacteria. Phagocytosis was indicated by increase in FITC signal as measured by flow cytometry. FITC uptake was observed within a 20 min period and we found approximately 20% gradual reduction of the FITC signal in SMSB4 treated samples compared to BSA treated samples (Figure 2a). We also measured FITC uptake in *S. aureus* pre-opsonized with heated serum and found a neglectable FITC signal (Figure 2a). These results indicate that (i) active complement was required to opsonize *S. aureus* and (ii) SMSB4 inhibits complement mediated-phagocytosis. The anti-phagocytic effect of the complement inhibitor SMSB4 is concentration dependent as the degree of inhibition increased with increasing concentration of protein used to treat the serum, e.g. from 30% inhibition at 25 µg/ml to 60% inhibition at 150 µg/ml of SMSB4 (Figure 2b).

#### (ii) SMSB4 reduces C3b deposition on *S. aureus* and C5 cleavage

**Figure 3:**
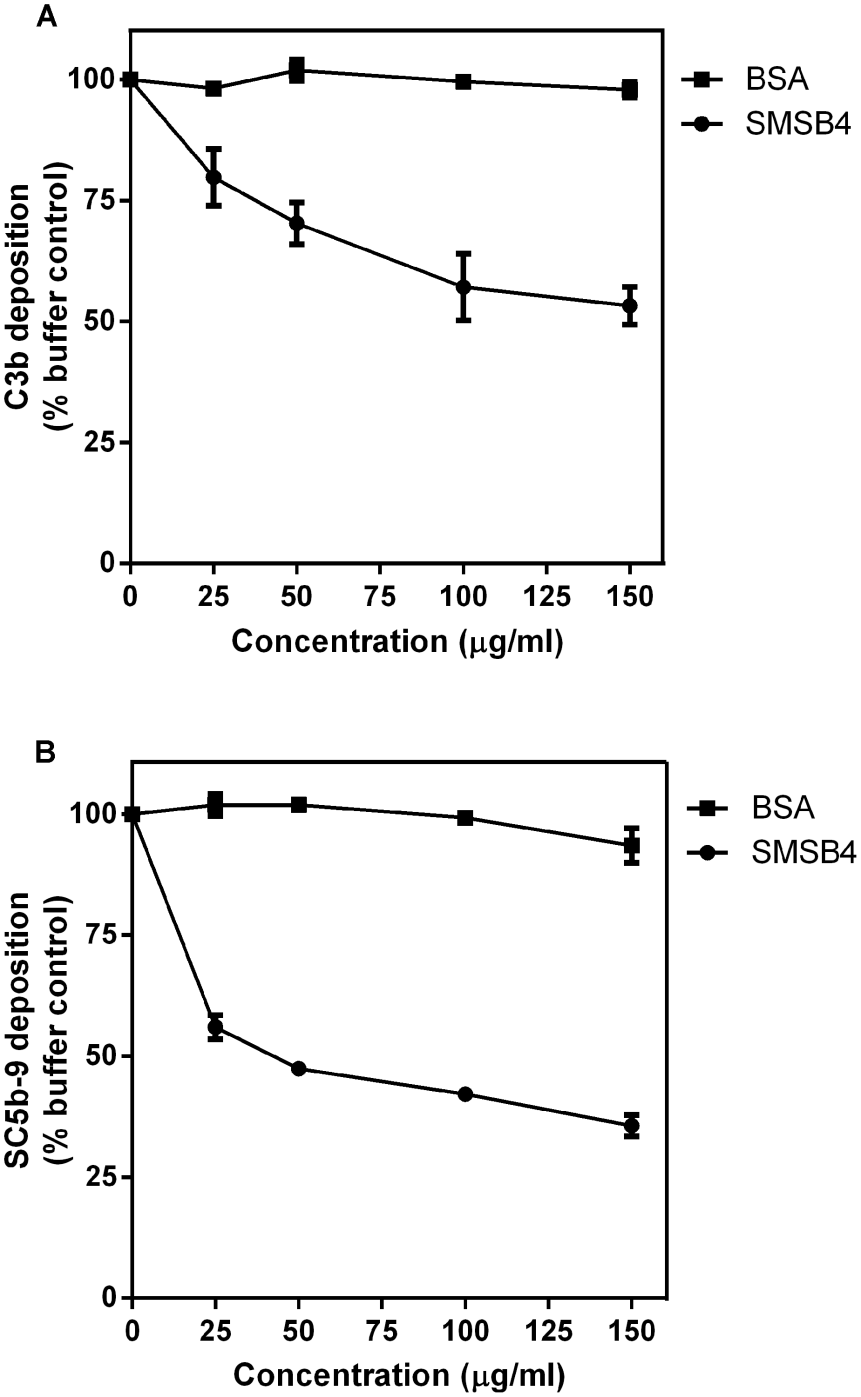
SMSB4 reduces deposition of C3b (A) and MAC complex (SC5b-9) formation (B). The wells of 96-well microtiter plates were coated with 100 µl aliquots of bacterial cell suspensions containing 5×10^6^ cfu/ml of *S. aureus*. Wells were then incubated with 10% NHS which has been pre-treated with increasing concentrations of either SMSB4 or BSA. Antibodies were detected by ELISA using primary human specific antibodies, followed by HRP-conjugated secondary antibodies, and fluorescence was detected at 490 nm. Results are shown as means ± SEM from three independent experiments.

Deposition of the complement component C3b on the pathogen surface, also known as opsonization, is crucial for phagocytosis. Opsonized bacteria are recognized by neutrophils via their cell surface receptor, leading to phagocytosis and killing of the pathogen. The effect of the mite complement inhibitor SMSB4 on the phagocytosis indicated that this molecule was likely to interfere with opsonization. *S. aureus* was incubated with NHS, pre-treated with various concentrations of SMSB4 or BSA as a negative control. Surface bound C3b was detected with a specific antibody. Our results demonstrate that in the presence of an increasing concentration of SMSB4, C3b deposition on *S. aureus* was reduced in a concentration dependent manner (Figure 3a), which was comparable to the degree of reduction in phagocytosis (Figure 2b).

In the complement cascade, the formation of C5 convertase (C4b2a3b/C3bBb3b) follows downstream of the C3 cleavage, arising from the binding of additional C3b onto C3 convertase on the bacterial surface. C5 convertase cleaves C5 into C5a and C5b. C5a is a potent chemoattractant to initiate migration of neutrophils towards the site of infection. C5b binds to other complement components (C6-C9) to form the MAC complex which is deposited on the surface of *S. aureus* [30]. To study whether SMSB4 affects C5 cleavage, we analyzed the amount of C5b deposited as part of the MAC complex on the *S. aureus* cell surface, using anti SC5b-9 antibody, a neoantigen-specific antibody recognizing the human MAC complex. We found that SMSB4 indeed reduced the level of MAC deposition on *S. aureus* in a concentration dependent manner (Figure 3b). We also noted that the degree of inhibition by SMSB4 on the MAC complex formation was much higher than its effect on the C3b deposition (Figure 3a). Since active C3 convertase and additional C3b are prerequisites for formation of C5 convertase, reduction in any of these components is expected have a profound effect on the C5 cleavage.

### SMSB4 inhibits the function of complement pathway initiations

**Figure 4:**
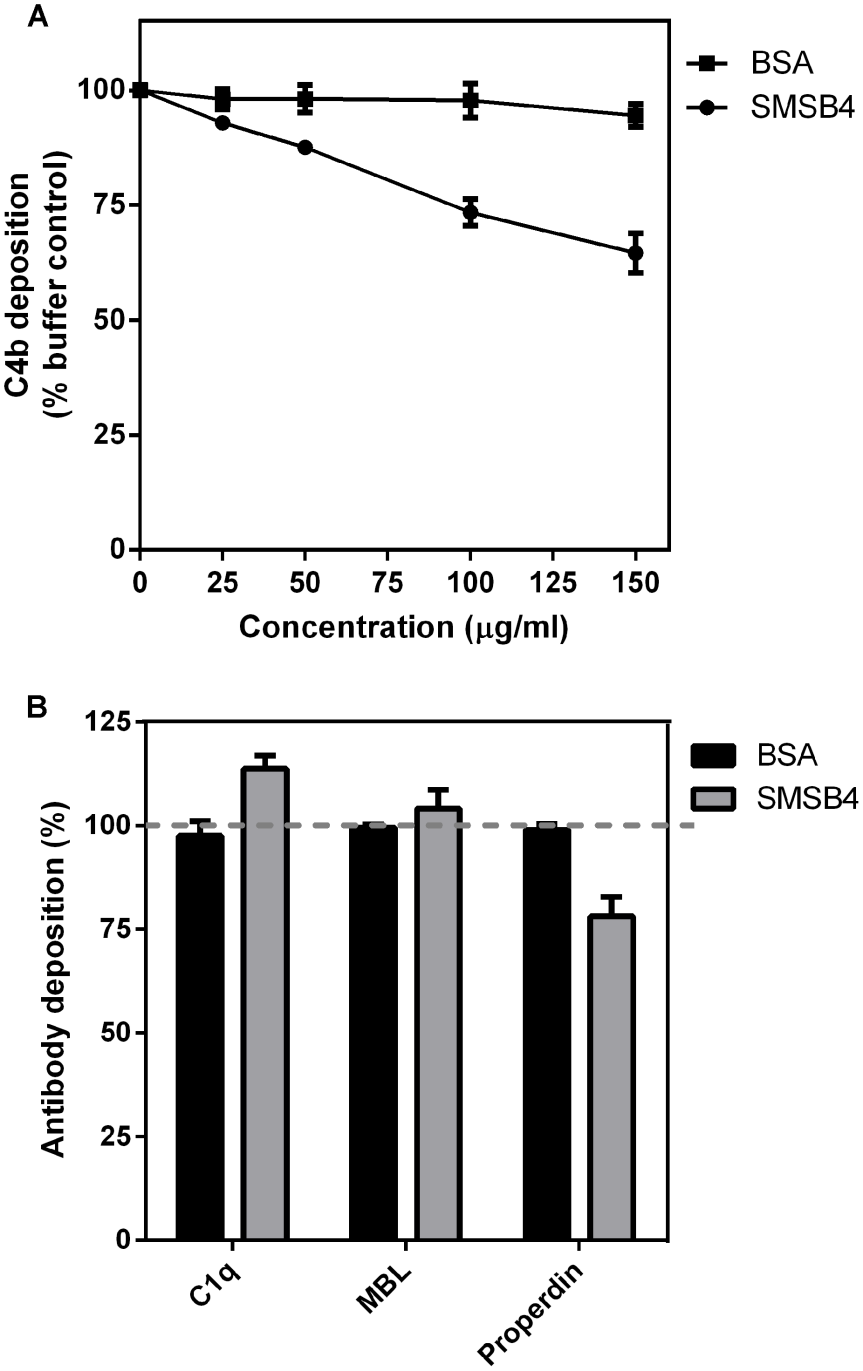
Effect of SMSB4 on the depositions of C4b (A), C1q, MBL and properdin (B) on *S. aureus* cells. The wells of 96-well microtiter plates were coated with 100 µl aliquots of bacterial cell suspensions containing 5×10^6^ cfu/ml of *S. aureus*. Wells were then incubated with 10% NHS which has been pre-treated with increasing concentrations of either SMSB4 or BSA. Antibodies were detected by ELISA using primary human specific antibodies, followed by HRP-conjugated secondary antibodies, and fluorescence was detected at 490 nm. Results are shown as means ± SEM from three independent experiments. The statistical significance of differences between BSA and SMSB4 treated samples were estimated using two way ANOVA with Sidak’s multiple comparison test. **, *p*<0.01; ***, *p*<0.001; ns, not significant (B).

Formation of C3 convertase via CP and LP requires cleavage of C4 by either C1 or MBL complexes, resulting in the covalent binding of C4b onto the bacterial surface. We previously reported that SMSB4 affects the initial and progressing steps of the complement activation for both the CP and the LP. Those assays were carried out in the absence of target cells by artificially activating the individual complement pathways with either IgG or mannan [25]. Here we investigated the effect of SMSB4 on the C4b deposition on the *S. aureus* cell surface when complement system is physiologically activated by the bacteria themselves. Our result showed that SMSB4 reduced the C4b deposition in a concentration dependent manner (Figure 4a).

Initiation of the complement cascades preceding C4 cleavage involves the deposition of C1q (CP), MBL (LP) and properdin (AP) onto the pathogen surface. We therefore examined whether SMSB4 affects the deposition of these complexes on *S. aureus* cell surface. SMSB4 did not affect the depositions of C1q and MBL. However, deposition of properdin was reduced by 20% in the samples treated with a 100 µg/ml SMSB4 (Figure 4b).

## Discussion

The complement system is an essential part of the innate immune system that rapidly recognizes and kills pathogen either by direct cell lysis, or marks the pathogen to be killed by phagocytes. Many pathogens, whether bacteria or parasites, are known to posses strategies to evade the host complement attack. For example, *S. aureus* is a versatile human pathogen that produces a diverse array of multiple complement inhibitors, which interfere with various stages of complement activation [20], [22], [23], [31], [32]. Scabies mites also produce a variety of proteins that interfere with complement [24]–[26]. We have localized these proteins in the mite gut and in fecal pellets by immunohistochemistry [25], [33]. In the confined space of the mite gut and epidermal burrows the combined anti-complement activity exerted by the multiple mite complement inhibitors may reach functionally and physiologically significant levels. For the mites as serum-feeding parasites, the biological relevance of scabies mite complement inhibitors is likely preventing complement-mediated mite gut damage [24], [25], [34]. We propose that these molecules also pre-condition the host for the onset of bacterial invasion. Epidemiological evidence supporting the frequent association of *S. pyogenes* and/or *S. aureus* co-infections with scabies infestations led to our hypothesis that scabies infection could promote the onset of infections by the opportunistic pathogenic bacteria. We previously reported that recombinant scabies mite complement inhibitors promoted the *in vitro* growth of *S. pyogenes* [26]. Here, we provide more detailed evidence of how one of these mite complement inhibitors, namely SMSB4, promoted the growth of *S. aureus*. This is the first report on the effect of a scabies mite complement inhibitor on aiding the growth of Staphylococcus.

Mechanism of complement-mediated killing is due to phagocytosis of bacteria by blood leucocytes and direct lysis by MAC complex. It is known that a gram-positive pathogen such as *S. aureus* is insensitive to direct killing by MAC-complex formation [30]. As such the inhibitory effect of SMSB4 on blood killing is likely due to the complement-dependent killing function of blood leucocytes. SMSB4 reduced opsonization, phagocytosis and effectively reduced the complement-mediated blood killing *of S. aureus*, and promoted the growth of the bacteria in a dose-dependent fashion. Furthermore SMSB4 also inhibited the generation of anaphylatoxins required for the phagocyte recruitment. Since complement response is a generalized immediate host immune reaction towards pathogens, we found that the inhibitory effect of SMSB4 on the bacterial killing was similar across all *S. aureus* strains tested, including MRSA and MSSA.

We previously shown that SMSB4 inhibits the initial and progressing steps of all three complement pathways [25]. In this previous work haemolytic, complement deposition and complement binding assays were employed, where the individual complement pathways were activated by antibody or carbohydrates. The effect on bacteria surfaces and neutrophils was not addressed. In the present study, we attempted for the first time to mimic pathophysiological conditions provided by the presence of bacteria by activating the complement cascades with *S. aureus* cells, and then analyzed the effect of SMSB4 on the cell surface deposition of complement complexes required for initialization of the complement cascades. We found that SMSB4 did not inhibit the deposition of C1 and MBL complexes on the bacterial surface. Our finding that SMSB4 reduced the amount of C4b deposition on the bacterial surface indicates that SMSB4 reduces the amount of C3 convertase (C4b2a) formation in the CP and LP pathways. Since C1 and MBL complexes are structurally and functionally homologous, both enzyme complexes cleaving C4, it is likely that SMSB4 interferes with the C4 protease function. Interestingly SMSB4 also reduced the amount of properdin deposited on the *S. aureus* cells. Properdin is required to stabilize AP C3 convertase (C3bBb), and hence a decreased level of properdin will decrease the formation of functional AP C3 convertase. Furthermore, properdin is reported to play a role in amplification of MBL-initiated LP [35], hence a reduction in AP will contribute to the overall complement activation. Indeed, we have previously established that SMSB4 reduces the C3b deposition via all three complement pathways [25]. This study provides additional data confirming that SMSB4 acts early on in the complement cascades and reduces the formation of functional C3 convertases, thereby inhibiting complement-mediated killing of the invading pathogen *S. aureus*. Further study is required to identify the exact mechanisms by which SMSB4 interferes with the individual complement components. We have assessed here the effect of one individual recombinant mite complement inhibitor under *in vitro* test conditions. It is possible that the amount of recombinant protein required to show effects in the assays presented does not exactly reflect the amounts of SMSB4 actually present in the *in vivo* situation. Further investigation will be required to determine the concentration and composition of complement-inhibitory properties in scabies infested skin. Moreover, since *S. aureus* is known to produce a wide arsenal of its own complement inhibitors, it cannot be ruled out that this bacterium may be beneficial to prolong the infestation by the scabies mite. Skin infestation with the mite *S. scabiei* has been documented as a primary risk factor for pyoderma and impetigo worldwide and treatment of scabies reduces the prevalence of bacterial infections [5], [6]. *S. aureus* has been isolated from epidermal mite burrows and fecal pellets [36] suggesting that mites themselves may contribute to the transmission of pathogenic bacteria. We now present evidence that secondary infection by *S. aureus* is in part a consequence of the SMSB4 driven suppression of opsonization and phagocytosis by phagocytes. Since scabies mites produce multiple families of complement inhibitors, it is tempting to speculate that collective action of complement inhibitors produced by scabies mites in a confined space of skin burrows either stop or delay the clearing effect of the immediate local innate immune response to pathogen invasion. This creates a window of opportunity for *S. aureus* to multiply and establish infection. Therefore scabies mite burrows in the epidermis may serve as a perfect microenvironment for both the mites and bacterial pathogens being shielded from the host innate immune system.
