# Correction: A Scabies Mite Serpin Interferes with Complement-Mediated Neutrophil Functions and Promotes Staphylococcal Growth

**DOI:** 10.1371/journal.pntd.0003415

**Published:** 2014-11-25

**Authors:** The PLOS Neglected Tropical Diseases Staff

Figure 1B and Figure 4 are incorrect because they do not show the updated data. The authors have provided the corrected versions here.

**Figure 1:**
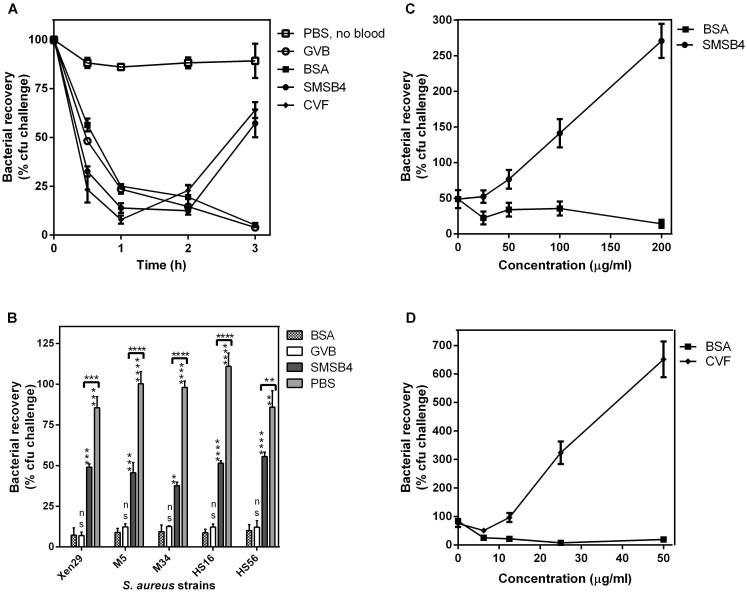
SMSB4 reduces the blood killing of *S. aureus* strain Xen29 in whole blood (A) and pyoderma isolates of *S. aureus* (B). SMSB4 promotes bacteria growth in a concentration dependent manner (C) similarly to CVF (D). *S. aureus* Xen29 or pyoderma isolates MRSA strains (HS16, M34), MSSA strains (HS56, M5) were harvested from mid-log growth phase culture. Bacteria (1×10^5^ cfu/ml) were challenged with whole blood pre-treated with either 100 µg/ml SMSB4, positive control 10 µg/ml CVF, negative controls 100 µg/ml BSA or GVB^2+^ buffer only. *S. aureus* cells in PBS only without blood was also included to illustrate that the reduction in bacteria number was due to blood killing (**A**). Numbers of bacteria were counted as cfu/ml at various time points (**A**) or at 3 h (**B**, **C**, **D**). Bacterial recovery was calculated as a percentage of the challenge dose. Results are shown as means ± SEM from three independent experiments. The statistical significance of differences between samples was estimated using two way ANOVA with Tukey’s multiple comparison test. **, *p*<0.01; ***, *p*<0.001; ****, *p*<0.0001, ns, not significant (B).

**Figure 4:**
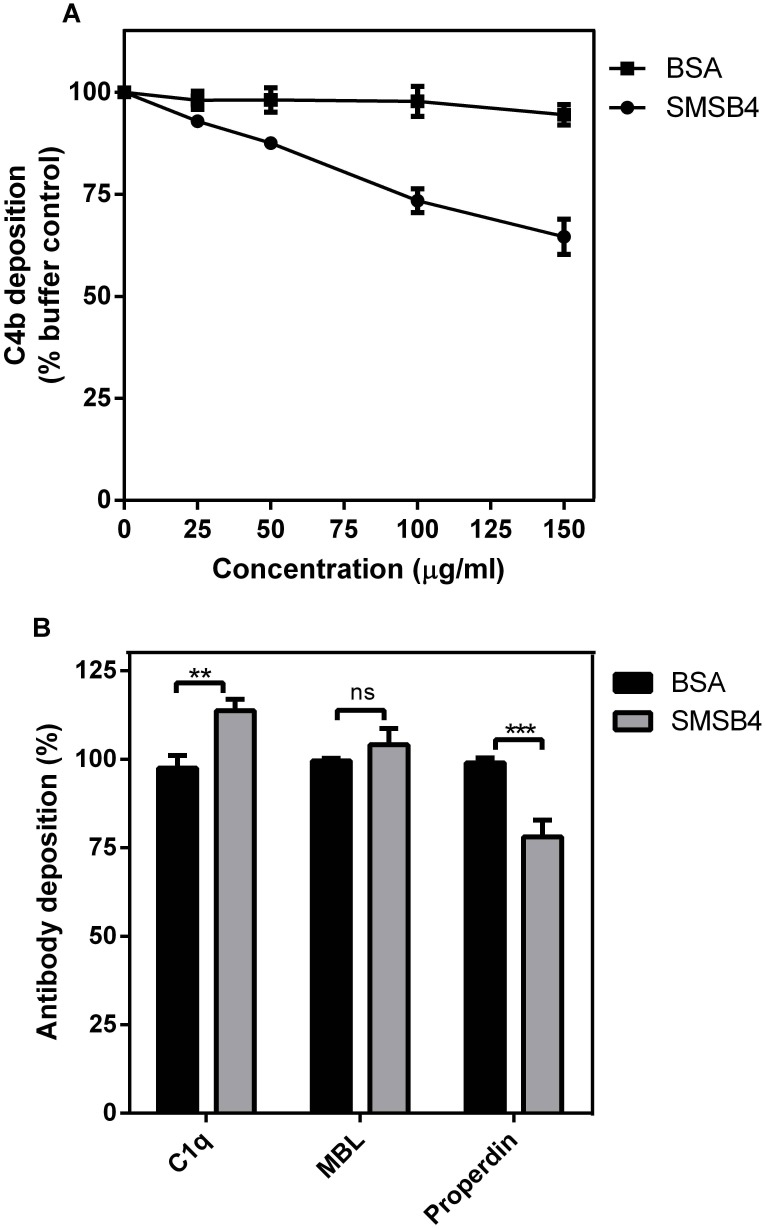
Effect of SMSB4 on the depositions of C4b (A), C1q, MBL and properdin (B) on *S. aureus* cells. The wells of 96-well microtiter plates were coated with 100 µl aliquots of bacterial cell suspensions containing 5×10^6^ cfu/ml of *S. aureus*. Wells were then incubated with 10% NHS which has been pre-treated with increasing concentrations of either SMSB4 or BSA. Antibodies were detected by ELISA using primary human specific antibodies, followed by HRP-conjugated secondary antibodies, and fluorescence was detected at 490 nm. Results are shown as means ± SEM from three independent experiments. The statistical significance of differences between BSA and SMSB4 treated samples were estimated using two way ANOVA with Sidak’s multiple comparison test. **, *p*<0.01; ***, *p*<0.001; ns, not significant (B).

## References

[1] Swe PM, Fischer K (2014) A Scabies Mite Serpin Interferes with Complement-Mediated Neutrophil Functions and Promotes Staphylococcal Growth. PLoS Negl Trop Dis 8(6): e2928 doi:10.1371/journal.pntd.0002928 24945501 10.1371/journal.pntd.0002928 PMC4063749

